# Public awareness, attitudes, behavior and norms building green hospitals' power

**DOI:** 10.1016/j.heliyon.2024.e39336

**Published:** 2024-10-18

**Authors:** Ari Nurfikri, Deni Danial Kesa, Mingchang Wu, Elsa Roselina, Abas Hidayat

**Affiliations:** a Vocational Education Program, Universitas Indonesia, Pondok Cina, Beji, Depok City, West Java, 16424, Indonesia; b National Yunlin University of Science and Technology, No. 123, Section 3, Daxue Rd, Douliu City, Yunlin County, 64002, Taiwan; c Sekolah Tinggi Ilmu Kesehatan Cirebon, Jl. Brigjend Dharsono No.12b, Kertawinangun, Kedawung, Cirebon, West Java, 45153, Indonesia

**Keywords:** Green hospital, Public awareness, Attitudes, Behavior, Norms

## Abstract

Hospitals function to provide health services to the community, but they have the potential to be contributors to hazardous waste, climate change, and global warming. The concept of green hospitals is now necessary for changing hospital functions developed by various hospitals worldwide. The success of the green hospital concept requires support from the perspective of the surrounding society. The research aims to analyze public awareness, attitudes, behavior, and norms to build the power of green hospitals. This research method is simple linear regression. The research instrument used a questionnaire. The respondents of this research were 400 green hospital users from Indonesia and Taiwan. The data analysis method includes tests for normality, linearity, and heteroscedasticity. Once these three tests are met, a linear regression analysis is conducted to understand the influence and prediction of public awareness, attitudes, behavior, and norms on building the power of green hospitals. The research results show that each aspect of public awareness, attitudes, behavior, and norms significantly positively influence the building of the power of green hospitals. Each additional value of public awareness, attitudes, behavior, and norms is expected to strengthen the image of a green hospital. Maintaining the sustainability of green hospital programs worldwide not only focuses on implementing the green concept but also needs to pay attention to public perception when choosing a hospital. The research contribution is significant to developing more sustainable green hospitals, increasing knowledge about green hospitals, and increasing public interest and perception of green hospitals.

## 1 Introduction

One of the efforts made to maintain the sustainability of human life worldwide is to build hospitals. The hospital continues to serve the public without stopping and experiences developments from time to time adapted to the needs of the hospital itself and the community. However, the world is currently experiencing a public and environmental health crisis. It has been proven that the industrialization of the world of health has the potential to produce pollution that can endanger local communities through energy consumption and waste accumulation resulting from operational activities [1]. According to Janik-Karpinska et al., over the last 30 years, health facilities, including hospitals, have produced a dramatically increasing amount of waste [2]. Odonkor & Mahami said that if health service waste management is not carried out properly, it can cause harm to humans and the environment [3]. Hospitals that initially functioned to provide health services to the public became contributors to hazardous waste and contributed to climate change and global warming.

The phenomenon of changing the function of hospitals, which initially was to provide health services to the community, has turned into the potential to cause a public health and environmental health crisis. The green hospital concept is emerging to overcome environmental challenges and meet public needs in health matters. Aini et al. argued that a green hospital is an environmentally friendly concept designed to empower existing natural potential as the leading resource [4]. It is environmentally friendly and meets current needs without reducing reserves for future needs. According to Tarkar, implementing green hospitals is expected to ensure environmental sustainability by reducing waste which negatively impacts the environment [5].

Green hospitals are now necessary for changing hospital functions developed by various hospitals worldwide. According to research by Shaabani et al., Iran has developed a standard green hospital model to maintain public health by reducing environmental risks [6]. Research by Sahamir et al., in Malaysia said that developing green hospitals is essential to create a healthy, economically, environmentally and socially feasible lifestyle [7]. In Taiwan, Shen said that the Taiwanese government is actively directing hospital management in the country towards the green hospital concept [8]. According to Ngatindriatun et al., green hospitals in Indonesia are a new trend in sustainable and environmentally friendly hospital management in construction planning, operations and maintenance [9].

Taiwan established a program in 2010 called “four emerging intellectual industries” that includes intelligent green buildings [10]. Green hospitals have adapted from the concept of green buildings. According to Gan et al., in Taiwan, green hospital projects are frequently viewed as a low priority among the numerous demands placed on busy hospitals [11]. However, these efforts can be a hospital's most vital contribution to patient care. As the destructive effects of climate change become more apparent, hospitals are taking an essential leadership role in attaining “climate-smart healthcare” and working toward a more sustainable future. However, the number of hospitals in Taiwan has expanded in response to rising home care demand, and many hospital systems work 24 h a day [12]. The government recommends the green hospital concept to lead to active changes in hospital management [8].

Indonesia advised all hospitals to implement green hospitals in 2020. Based on the Republic of Indonesia Minister of Health regulation no. 7 of 2019, concerning hospital environmental health, states that a hospital is a health service facility, a gathering place for sick and healthy people, or can be a place for disease transmission and allows environmental pollution and health problems to occur [13]. Therefore, organizing a hospital's ecological health by health requirements is necessary. Hospitals need to fulfil the principles of green hospitals to create hospitals that are anticipatory towards environmental pollution, resource efficiency, and the impact of global warming and climate change [14].

Currently, many countries are developing the concept of green hospitals. This can be seen from the many members of the international network called Global Green and Healthy Hospitals (GGHH), with the aim that hospitals, health service facilities and health organizations must be dedicated to reducing negative environmental impacts. GGHH has over 1,900 members from 86 countries [15]. Therefore, the research took samples from two GGHH member countries: Indonesia and Taiwan.

The target of this study is to measure and predict the value of public awareness, attitudes, behavior, and norms on building the power of green hospitals. The study is expected to contribute significantly to developing more sustainable green hospitals, increasing knowledge about green hospitals, and increasing public interest and perception of the image of green hospitals. The research plan will conduct a literature review on green hospitals, public awareness, attitudes, behavior, and norms. Furthermore, the researchers developed a questionnaire to collect data on the understanding of green hospitals, aspects of public awareness, attitudes, behavior, and norms. The researchers used statistical analysis to interpret the data obtained from the survey. The researchers looked for correlations between awareness, attitudes, and behavior toward the power of green hospitals. Finally, the researchers predicted green hospitals' power from the perspective of public awareness, attitudes, behavior, and norms.

### 1.1 Green hospital

Green hospitals have adapted from the concept of green buildings. Green building principles are transforming building practices in response to growing concerns about pollution and environmental damage, increasing awareness and acceptance of climate change, diminishing resources, rising energy costs, and growing demand for sustainability in the construction and design of buildings [16].

A green hospital is a hospital that improves public health by continuing to reduce its environmental impact on sustainability [5]. Environmentally friendly hospitals need to be designed to utilize the potential of the natural environment efficiently. It is also necessary to pay attention to waste generated from hospitals, especially hazardous waste (biohazards), which must be destroyed by burning at temperatures above 800 °C. Another important thing is to create good air circulation and lighting, which can be achieved using natural lighting and ventilation [14].

The green hospital concept is carried out to minimize the hospital's contribution to global warming, reduce the impact of environmental damage, and improve air quality with natural ventilation [17]. Apart from that, the importance of open space around the hospital, proper water management, healing gardens, comfortable accessibility for hospital users, and good management of medical and non-medical waste [18].

The green hospital design concept can be implemented through the use of materials that are durable, not dangerous, and easy to maintain. The choice of building materials should be domestically produced to lower the resulting gas emissions compared to imported materials. It is also hoped that environmentally friendly construction that considers several natural aspects, such as sunlight, natural ventilation, alternative energy, and so on, can be implemented [19].

Energy savings in design can be achieved by using ventilation/voids to utilize sunlight and air from outside the building and using energy-saving lamps. Meanwhile, water savings can be achieved using an automatic tap, saving more water than a manual tap [20]. In terms of construction, it is necessary to select environmentally friendly materials (nontoxic) that can reduce noise. Efficient use of artificial resources in hospitals by controlling electricity use can also help reduce environmental impacts. There is also a need for rainwater collection containers around the hospital environment to reuse wastewater and harvest rainwater [14].

The importance of environmental management can be pursued in implementing the green hospital concept [21]. This concept is done to create a healthy environment by creating a hospital environment that is free of cigarette smoke and adding green spaces to improve the air quality in the hospital. Excessive energy use is also a factor in the greenhouse effect, so saving on using artificial energy is also necessary. Another step that can be implemented to preserve the hospital environment is to manage the waste produced appropriately and correctly and save water use [22].

Green hospitals are important for several reasons: 1) Implementing environmentally friendly practices can reduce exposure to hazardous substances for patients and staff, creating a healthier environment [23]. 2) Green hospitals focus on better waste management, including recycling and reducing medical waste, which helps reduce environmental impact [24]. 3) Using energy-efficient technologies reduces energy consumption and operating costs, providing long-term financial benefits [25]. 4) Raising awareness of environmental issues among patients and staff can lead to broader green behaviors [26]. 5) Green hospitals also support overall community health by contributing to environmental health [27]. 6) Being an environmentally friendly hospital can enhance a hospital's reputation and attract patients who care about environmental issues [28].

Green hospitals promote health and well-being through environmentally friendly and sustainable practices. However, there are problems and challenges faced in green hospitals, including high initial costs for green technologies [29] because profitability issues are always a major issue in hospital management [30], sustainable medical waste management [31], compliance with complex environmental regulations, lack of education and awareness among staff and patients [28], and difficulties in changing existing habits and infrastructure [32]. In addition, support from all stakeholders is also essential for the success of green initiatives. Addressing these problems and challenges requires an approach that involves the perception of the community as hospital users to overcome stigma and improve access to accurate health information.

Several previous researchers have studied the sustainability of green hospital implementation. Annah et al. discuss how green hospitals help increase public knowledge about the importance of protecting the environment for 100 respondents. This research shows that green hospitals positively impact the development of brand certification and brand image as a mediator of patient trust [33]. Afifi & Amini researched green hospitals to create customer loyalty among 209 respondents [34]. The implications of this research show that the basic concept of green hospital service quality is essential to increasing customer loyalty and customer value in green hospital management. The research of Tarkar states that the green hospital concept is a concept that is worthy of being exemplified and implemented because it provides social, economic and environmental benefits [5]. Research by Shih et al., said that a green hospital is sufficient in power, with a handy interface and ease of use; the staff would then feel that it is helpful for their work [35]. Research by Billanes et al. found that green hospitals, based on evidence in Philippine hospitals, can increase occupant comfort, reduce energy consumption and carbon emissions, and maintain energy reliability with intelligent technology [36]. Research by Mashayekhi Mazar et al. found that the laboratory, laundry, and kitchen conditions, water management, pollutant management and dissemination to the air, and environmental strategies for meeting green hospital requirements by ISO 14000 are appropriate and optimal [37].

Previous studies have discussed the relationship between implementing the green hospital concept and knowledge, marketing, economy, social, and environmental management. As a novelty, this research is different from previous research; this research links green hospitals to public awareness, attitudes, behavior and norms. Supported by Soqia et al., hospitals as public facilities need to be supported by public awareness methods [38].

### 1.2 Public awareness, attitudes, behavior and norms

There are various types of hospitals worldwide, both managed by the private and government. The increasing number of hospitals will indirectly increase their competitiveness. Amid hospital competition, management innovation is needed to provide excellent service and a good image in society. Hospitals without advantages and strengths cannot compete, which results in being abandoned by their patients. Soqia et al. said that hospitals as public facilities need to be supported by community awareness methods [38]. However, public awareness of excellent service and hospital image is only sometimes a reason for choosing a hospital. In cases where patients require urgent treatment, the patient's family prefers a hospital, considering the distance. Public considerations in selecting a hospital apart from the urgent patient situation must also be considered. Patients receiving outpatient or inpatient treatment need to be given awareness as a step for hospital management to increase competitiveness and maintain hospital sustainability.

Society, with its various characteristics, is very selective in choosing hospital health services and other health services. The higher the public's awareness of hospital services, the more selective they will choose a hospital. Public awareness regarding the importance of health influences hospitals in providing health services. In this era of global warming, public awareness of the environmental impacts caused by the activities of buildings, factories and other facilities, including health facilities. Evidence from findings by Almulhim shows that 79.2 % of people are worried about the harmful impacts of pollution and hope there are other alternatives so that the activities carried out do not damage the environment [39]. Public awareness that the environment is in danger due to climate change caused by humans and natural phenomena is one of the reasons for utilizing the green concept in buildings, factories and other facilities, including health facilities.

The public's attitudes towards something new, both from within and outside society, influence the acceptance of something. According to Hornsey & Fielding, many people agree and accept that humans are one of the causes of climate change [40]. Therefore, the world community has responded positively to the innovation of the concept of buildings, factories and facilities that maintain the sustainability of the surrounding environment. Society's acceptance of the world's current climate conditions is essential in choosing and considering things related to their impact on the surrounding environment.

Public behavior is caused by the level of public awareness and attitude towards something when choosing and deciding on an action. The causes of bad behavior are a lack of public awareness and negative attitudes [41]. People's behavior of doing things that do not damage the environment is the basis for choosing something to use or consume [42]. In hospital selection, people's behavior in choosing a hospital is based on something other than expensive costs and sophisticated equipment. However, good waste management and environmental friendliness are determining factors for consumers when making choices [43].

Norms are social rules or benchmarks for human behavior in social life. Public norms are the rules that regulate the behavior of community members in interacting with each other. According to Cialdini & Jacobson, maintaining environmental sustainability is a vibrant focus area for studying societal norms [44]. Through a communication approach, individuals carry out norms applicable in society in disseminating information. Community habits in disseminating information have become norms for communication between individuals, families, organizations and society, both directly and through the media. Habits of disseminating information without realizing it can impact society's response to something. In the era of global warming, reducing negative environmental impacts and maintaining sustainability is undoubtedly important news in society. In the context of hospital selection, it makes it easier to spread the news about hospitals that use environmentally friendly and sustainable patient care equipment, such as non-toxic cleaning products and environmentally friendly medical equipment.

Promoting public awareness, attitudes, behavior, and norms towards green hospitals is important to improve service quality, build better relationships with the community, and create an environment that supports better health. According to Prang et al., public awareness of the quality of hospital services and facilities is important to help patients make better decisions [45]. According to Liu et al., positive attitudes toward a hospital's reputation are important because they can increase patient confidence in choosing a place of care [46]. According to Moscone et al., people select hospitals that are considered reasonable based on recommendations and social norms, which influence their decisions [47]. Norms about health care can influence individual choices, such as preferences for certain hospitals or alternative treatments.

## 2 Materials and methods

This research method is simple linear regression. The regression method aims to analyze public awareness, attitudes, behavior and norms to build the power of green hospitals. This method is a solution to test statistical hypotheses and predict the magnitude of the influence of the variables of public awareness, attitudes, behavior and norms increased or decreased on the power of green hospitals.

The minimum sample size selected using Lemeshow formula [48], with a maximum estimate of 50 % and an error rate of 5 % for a population size that is not known with certainty, is 384. Based on this minimum sample, the researchers selected 400 respondents. The respondents of this research were 400 green hospital users from Indonesia and Taiwan. The sample selection strategy uses a purposive sampling approach through inclusion criteria, with the requirements being outpatients of green hospitals aged 18–55 who understand the concept of green hospital and environmental problems. Purposive sampling was decided based on established criteria to ensure the study could proceed without potential bias.

The research instrument uses a Likert scale questionnaire with a scale of 1–5 for each statement [49]. Recapitulation of answers is carried out by converting Likert scale values into percentages for each answer. Statements about green hospitals were selected and adapted based on the green hospital from the concept in articles by Billanes et al. [36] and Tarkar [5]. Statements about public awareness and behavior were adapted from the concept in articles by Psillaki et al. [50], Soqia et al. [38], and Hornsey & Fielding [40]. Statements about norms in communication in society is adapted from the concept in articles by Khoong et al. [51]. Table 1 below is an indicator of the questionnaire statement.

**Table 1:** Indicators for questionnaire statements.

Variables	Indicators	
	No	Statements
The power of green hospital (Y)	1.	The concept of a green hospital made me aware of the importance of hospitals that maintain the sustainability of the surrounding environment.
	2.	The concept of a green hospital makes me more accepting of hospitals that maintain the sustainability of the surrounding environment.
	3.	The concept of a green hospital makes me prefer hospitals that maintain the sustainability of the surrounding environment.
	4.	The concept of a green hospital has become my benchmark for sharing information in deciding on a hospital.
Public awareness (X1)	5.	I realize that hospitals which implement green hospitals will help protect the surrounding environment
	6.	I realize that hospitals that have implemented green hospitals can reduce negative impacts on the surrounding environment due to high energy consumption and waste generation.
	7.	I realize that hospitals that apply the green concept can improve the hospital's image.
Attitudes (X2)	8.	I am more accepting of health services at a green hospital focusing on reducing energy consumption and promoting renewable energy sources.
	9.	I am more accepting of health services at a green hospital that emphasizes creating and maintaining green spaces within the hospital premises, including gardens and green roofs, to enhance the healing environment.
	10.	I am more accepting of health services in hospitals that emphasize using environmentally friendly and sustainable patient care equipment, such as non-toxic cleaning products and environmentally friendly medical equipment.
Behavior (X3)	11.	I chose health services at a green hospital that focuses on reducing energy consumption and promoting renewable energy sources.
	12.	I chose health services at a green hospital that emphasizes creating and maintaining green spaces within the hospital premises, including gardens and green roofs, to enhance the healing environment.
	13.	I choose health services in hospitals that emphasize using environmentally friendly and sustainable patient care equipment, such as non-toxic cleaning products and environmentally friendly medical equipment.
Norms (X4)	14.	I get the influence of information from family, neighbours, and friends when deciding to use hospital services that have implemented the green hospital concept.
	15.	I get the influence of information from mass media or social media in deciding to use hospital services that have implemented the green hospital concept.
	16.	I get the influence of information from community groups, community organizations, or environmental organizations when deciding to use hospital services that have implemented the green hospital concept.

To ensure the accuracy and reliability of statements adapted from several articles, the instruments in Table 1 have been assessed using a reliability scale. Based on calculations with the help of SPSS software, Cronbach's Alpha reliability value is 0.832. According to the Guilford reliability criteria [52], this value is included in the high-reliability category. These results mean that the instruments in Table 1 can be used to make accurate and valid analyses in this research.

Based on the variables in Table 2, four simple linear regression models can be formulated: 1) Y and X1; 2) Y and X2; 3) Y and X3; 4) Y and X4. The authors proposed four statistical hypotheses (H) based on the four simple linear regression models. H1: Public awareness significantly and positively influences building green hospitals' power. H2: Public attitudes significantly and positively influence building green hospitals' power. H3: Public behavior significantly and positively influences building green hospitals' power. H4: Public norms significantly and positively influence building green hospitals' power.

**Table 2:** Results of the Kolmogorov-Smirnov normality test.

	Variables				
	Y	X1	X2	X3	X4
N	400	400	400	400	400
Asymp. Sig. (2-tailed)	0.067	0.054	0.058	0.062	0.066

The data analysis method was conducted in two stages. First, the researchers conducted normality, linearity, and heteroscedasticity tests to determine the suitability of the data obtained for linear regression analysis. Second, after the three tests were met, the researchers conducted a simple linear regression analysis with the target of understanding the significance of the influence between one variable and another through hypothesis testing, conducting linear equation modeling, and predicting public awareness, attitudes, behavior, and norms on building the power of green hospitals.

## 3 Results

### 3.1 Normality

This study conducted a normality test for data suitability before a simple linear regression analysis. The number of respondents in this study was >100. Therefore, the Kolmogorov-Smirnov test was used. Table 2 below shows the results of the Kolmogorov-Smirnov test.

Table 2 shows the value of Asymp. Sig. (2-tailed) variables Y = 0.067, X1 = 0.054, X2 = 0.058, X3 = 0.062 and X4 = 0.066. These values are greater than the sig criterion. 0.05, then the data for variables Y, X1, X2, X3 and X4 have a normal distribution. Based on these results, this research data meets simple linear regression analysis requirements.

### 3.2 Linearity

This study conducted a linearity test for data suitability before a simple linear regression analysis. Linearity criteria if the value of Sig. Deviation from Linearity >0.05 means the data has a linearity relationship. Table 3 below shows the results of the linearity test for each regression model.

**Table 3:** Results of linearity test.

Deviation from Linearity	Variables	Value of Sig.
	Y∗X1	0.515
	Y∗X2	0.614
	Y∗X3	0.682
	Y∗X4	0.673

Table 3 shows that the regression models Y and X1 have a value of sig. 0.515, the Y and X2 regression models have a value of sig. 0.614, the Y and X3 regression models have a value of sig. 0.682, and the Y and X4 regression models have a value of sig. 0.673. These values are more significant than the Sig linearity criterion >0.05. It can be concluded that there is a linearity relationship in the models Y and X1, Y and X2, Y and X3, Y and X3. Based on these results, this research data meets simple linear regression analysis requirements.

### 3.3 Heteroscedasticity

**Fig. 1:**
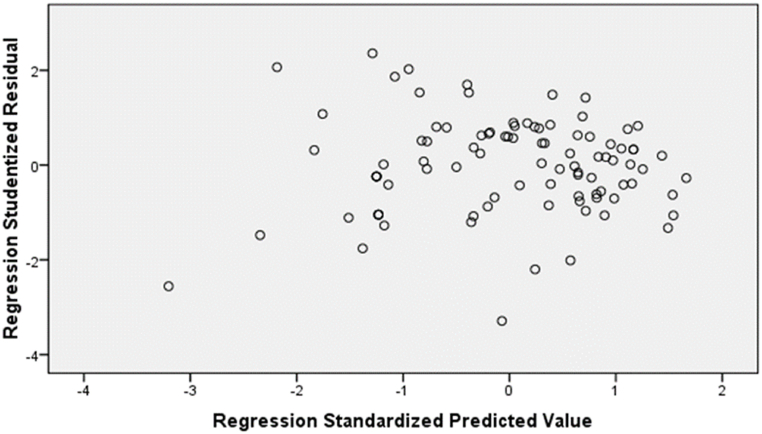
Scatterplot.

This study used a heteroscedasticity test for data suitability before conducting a simple linear regression analysis. In this study, a heteroscedasticity test was carried out by looking at patterns in Scatterplots [53]. Fig. 1 below is the SPSS software output in the form of scatterplots.

Fig. 1 shows the distribution pattern of points on scatterplots. As a result of the authors' observations, it is known that 1) The data points spread above and below or around 0. 2) The points do not gather only above or below. 3) The distribution of data points does not form a wavy pattern that widens, then narrows and widens again. 4) The distribution of data points is not patterned. Based on observations, the authors concluded that there was no heteroscedasticity problem. Therefore, it meets the requirements for an ideal regression model analysis.

### 3.4 Simple linear regression analysis

The results of normality, linearity, and heteroscedasticity tests show that the requirements for carrying out simple linear regression analysis have been met. There are four simple linear regression models to prove how much value public awareness, attitudes, behavior, and norms have for building the power of green hospitals. Apart from that, the four hypotheses proposed in this research must be answered. Table 4 below shows the data processing results using SPSS software by looking at the output regression coefficient values.

**Table 4:** Coefficient of regression.

Model	Variables	Unstandardized Coefficients		Sig
		B	Std. Error	
1	(Constant)	10.854	1.986	
	X1	0.839	0.025	0.000
2	(Constant)	20.213	2.391	
	X2	0.731	0.030	0.000
3	(Constant)	18.328	2.523	
	X3	0.735	0.031	0.000
4	(Constant)	21.541	2.388	
	X4	0.703	0.030	0.000

a. Dependent Variable: Y.

Based on Table 4, the results of regression model 1 show that the constant value of Unstandardized Coefficients is 10.854, and the value of public awareness (X1) is 0.839. So, the linear regression equation obtained for model 1 is Y = 10.854 + 0.839X. This means that if the value of the public awareness variable is zero, then the green hospital variable is 10,854. The regression coefficient of 0.839 states that every increase in the value of the public awareness variable will increase the strength of the green hospital. This means that every increase in public awareness positively influences the strength of a green hospital, which contributes to maintaining the sustainability of the surrounding environment. The significant value of X1 is 0.000, indicating it is less than the sig criterion value. 0.05, then hypothesis 1 for regression model 1 in this study is accepted. This means that public awareness positively influences building the power of green hospitals.

Based on Table 4, the results of regression model 2 show that the constant value of Unstandardized Coefficients is 20.213, and the value of public attitudes (X2) is 0.731. So, the linear regression equation obtained for model 2 is Y = 20.213 + 0.731X. This means that if the value of the public attitude variable is zero, the green hospital variable is 20,213. The regression coefficient of 0.731 states that each increase in the value of the public attitude variable will increase the strength of the green hospital. This means that every additional public attitude positively influences the strength of a green hospital, which contributes to maintaining the sustainability of the surrounding environment. The significant value of X2 is 0.000, indicating it is less than the sig criterion value. 0.05, then hypothesis 2 for regression model 2 in this study is accepted. This means that public attitudes positively influence building the power of green hospitals.

Based on Table 4, the results of regression model 3 show that the constant value of Unstandardized Coefficients is 18.328, and the value of public behavior (X3) is 0.735. So, the linear regression equation obtained for model 3 is Y = 18.328 + 0.735X. This means that if the value of the public behavior variable is zero, then the green hospital variable is 18,328. The regression coefficient of 0.735 states that every time there is an increase in the value of the public behavior variable, it will increase the strength of the green hospital. This means that every additional public behavior positively influences the strength of a green hospital, which contributes to maintaining the sustainability of the surrounding environment. The significant value of X3 is 0.000, indicating it is less than the sig criterion value. 0.05, then hypothesis 3 for regression model 3 in this study is accepted. This means that public behavior positively influences building the power of green hospitals.

Based on Table 4, the results of regression model 4 show that the constant value of Unstandardized Coefficients is 21.541, and the value of public norms (X4) is 0.703. So, the linear regression equation obtained for model 4 is Y = 21.541 + 0.703X. This means that if the value of the public norm variable is zero, then the green hospital variable is 21,541. The regression coefficient of 0.703 states that every time there is an increase in the value of the public norm variable, it will increase the strength of the green hospital. This means that every addition to public norms positively influences the strength of a green hospital, which contributes to maintaining the sustainability of the surrounding environment. The significant value of X4 is 0.000, indicating it is less than the sig criterion value. 0.05, then hypothesis 4 for regression model 4 in this study is accepted. This means that public norms positively influence building the power of green hospitals.

## 4 Discussion

### 4.1 Public awareness and the power of green hospital

The results of simple linear regression analysis in this study show that each additional value of public awareness increases the strength of the green hospital image, and public awareness positively influences building the power of green hospitals. Forming public awareness about the importance of protecting the environment to support the sustainability of the green hospital program. From a marketing perspective, consumer awareness about the product's superiority will attract them to return [54,55]. Likewise, in implementing the green hospital concept, public awareness becomes a force that attracts patients to visit again. Regression analysis of this research's results shows that the coefficient of regression is 0.839, meaning that every additional public awareness has a positive influence on increasing the strength of green hospitals. In contrast to the results of a cross-sectional survey conducted by Aryankhesal and Sheldon in Iran involving 104 outpatients across eight hospitals, patients emphasized hospital reputation as a primary factor. Nonetheless, developing an assessment of hospital performance necessitates awareness in selecting health services [56].

Kumari & Kumar say firmly that green hospitals are not an option but a necessity [16]. However, there needs to be public awareness regarding the benefits of green hospitals on environmental health to achieve this. Public awareness to choose environmentally friendly health facilities. Public awareness that waste from health facilities can cause environmental problems. Sigalingging et al. said that the public must be aware that when using health facilities, they must pay attention to the management of these facilities when anticipating environmental pollution, resource efficiency, and the impact of warming and global climate change [14].

### 4.2 Public attitudes and the power of green hospital

The results of simple linear regression analysis in this study show that each additional public attitude value increases the strength of the green hospital image, and public attitudes positively influence building the power of green hospitals. From a marketing perspective, according to Mehta & Chahal, academics need to comprehensively understand the profile of environmentally friendly consumers to foster consumer attitudes and willingness to use [57]. Likewise, when implementing the green hospital concept, public attitudes are essential to foster a willingness to use the hospital. Regression analysis of this research's results shows that the coefficient of regression is 0.731, meaning that every additional public attitude has a positive influence on increasing the strength of green hospitals. In line with the survey by Vonberg et al. involving 1000 households in Germany, the reputation of health service facilities was found to be irrelevant, whereas consumer attitudes play a significant role in hospital selection [58]. The green hospital program will run well with a positive attitude from the public.

Public attitudes towards acceptance of hospital management are one of the factors in the success of the green hospital concept. Hospital management, for example, focuses on reducing energy consumption and promoting renewable energy sources [50]. Hospitals emphasize the creation and maintenance of green spaces within the hospital environment, including gardens and green roofs, to enhance environmental healing [59]. Hospitals emphasize using environmentally friendly and sustainable patient care equipment, such as non-toxic cleaning products and environmentally friendly medical equipment [60]. Public attitudes towards accepting green hospitals are essential for implementing and developing the green hospital concept.

### 4.3 Public behavior and the power of green hospital

The results of simple linear regression analysis in this study show that each additional public behavior value increases the strength of the green hospital image, and public behavior positively influences building the power of green hospitals. From a marketing perspective, behavior influences consumer interest in repurchasing products [61,62]. Like implementing the green hospital concept, public behavior is essential to foster a willingness to revisit the hospital.

People's behavior toward doing things that are good for the environment is why consumers experience difficulty achieving responsible and sustainable consumption [42]. Patients, as hospital users or consumers, have the behavior of choosing a hospital for specific reasons, such as cost, brand, etc. Regression analysis of this research's results shows that the coefficient of regression is 0.735, meaning that every additional public behavior has a positive influence on increasing the strength of green hospitals. In line with case study in Indonesia by Wiwik Suryandartiwi & Abdul with 100 respondents, the behavior of choosing a hospital is caused by the intention to choose [63]. A case study conducted in Iran by Ravangard et al. supports this research. From a study of 330 respondents, it was found that behavioral control is one of the factors in the public sector that influences patients' intention to use healthcare [64]. Public knowledge about hospitals that focus on reducing energy consumption and promoting renewable energy sources gives rise to positive attitudes, intention to visit and behavior in choosing that hospital.

### 4.4 Public norms and the power of green hospital

The simple linear regression analysis results in this study show that each additional value of public norms increases the strength of the green hospital image, and public norms positively influence the building of the power of green hospitals. From a marketing perspective, market competition needs to pay attention to the norms that apply in society [65]. In the same way, implementing the green hospital concept must align with public norms to foster a willingness to visit the hospital.

According to Cialdini & Jacobson, maintaining environmental sustainability is a rich focus area for studying societal norms [44]. In principle, norms are external rules that determine normal and acceptable standards of behavior in everyday interactions, for example, digital social communication norms [66]. Regression analysis of this research's results shows that the coefficient of regression is 0.703, meaning that every additional public norm has a positive influence on increasing the strength of green hospitals. In line with Cham et al.'s research, a case study of 294 Chinese medical tourists using structural equation modeling revealed that social media communication influences the spread of hospital brand image and serves as a determinant in hospital choice [67]. People's habits in disseminating digital information have become communication norms between individuals, families, organizations and society. Communication habits make spreading news about hospitals that use environmentally friendly and sustainable patient care equipment easier. This approach positively impacts the image of a green hospital to foster a willingness to visit the hospital.

## 5 Conclusion

The green hospital concept is an approach to overcoming environmental challenges and meeting public needs in health matters. The surrounding community's perspective is a supporting factor in the sustainability of green hospital implementation. The researchers' basic assumption is that the supporting factors for the sustainability of green hospital implementation from the community perspective, such as public awareness, attitudes, behavior, and norms, contribute to strengthening and developing green hospitals in a sustainable manner. The results of simple linear regression analysis show that each additional value of public awareness, attitudes, behavior and norms strengthens the image of a green hospital. The results of hypothesis testing show that each aspect of public awareness, attitudes, behavior, and norms significantly positively influences the building of green hospitals' power. It is hoped that the empirical evidence from this research will provide information that to maintain the sustainability of the green hospital program throughout the world, it is not only necessary to focus on implementing the green concept but also needs to pay attention to public perceptions when choosing a hospital as one of the marketing strategies to introduce the green hospital concept.

The practical implication underlines this research's novelty in applying the green hospital concept, which involves more than just constructing eco-friendly hospital facilities and implementing environmentally sustainable practices. It necessitates viewing it from the public's perspective. From the public's perspective, patient satisfaction is very important. Still, it is equally important to raise awareness about how eco-friendly hospitals can protect the environment and reduce the negative impact of hospital waste. It is essential to consider community attitudes towards green hospital services, focusing on reducing energy consumption, using environmentally friendly patient care equipment, and incorporating green spaces within hospital premises. Understanding people's behaviors in selecting healthcare services and respecting public norms in receiving information about the benefits of green hospitals are equally important. The synergy between green practices and public perception is expected to enhance the effectiveness of implementing green hospitals.

This study's limitation is that it uses a simple regression analysis method, but further research can be developed using a multiple regression analysis method. This study uses the perspective of the community as users of hospital services, and further research can be developed using the perspective of hospital managers in an effort to draw strength from the existence of green hospitals.

## CRediT authorship contribution statement

**Ari Nurfikri:** Writing – review & editing, Writing – original draft, Visualization, Validation, Supervision, Resources, Project administration, Methodology, Investigation, Formal analysis, Data curation, Conceptualization. **Deni Danial Kesa:** Writing – review & editing, Writing – original draft, Visualization, Validation, Supervision, Methodology, Investigation, Formal analysis, Data curation, Conceptualization. **Mingchang Wu:** Writing – review & editing, Writing – original draft, Visualization, Validation, Supervision, Methodology, Investigation, Formal analysis, Data curation, Conceptualization. **Elsa Roselina:** Writing – review & editing, Writing – original draft, Visualization, Validation, Supervision, Project administration, Investigation, Formal analysis, Data curation, Conceptualization. **Abas Hidayat:** Writing – review & editing, Writing – original draft, Visualization, Validation, Supervision, Software, Methodology, Investigation, Formal analysis, Data curation, Conceptualization.

## Declaration of competing interest

The authors declare that they have no known competing financial interests or personal relationships that could have appeared to influence the work reported in this paper.
